# Supplementary material for: Photodynamic Therapy of Aluminum Phthalocyanine Tetra Sodium 2-Mercaptoacetate Linked to PEGylated Copper–Gold Bimetallic Nanoparticles on Colon Cancer Cells
Source: Int J Mol Sci. 2023 Jan 18;24(3):1902. doi: 10.3390/ijms24031902 (PMC9915188; doi:10.3390/ijms24031902)
Supplement: Supplementary file 1 [file ijms-24-01902-s001.zip › ijms-2045015-supplementary.pdf]

# Photodynamic Therapy of Aluminum Phthalocyanine Tetra Sodium 2-Mercaptoacetate Linked to PEGylated Copper–Gold Bimetallic Nanoparticles on Colon Cancer Cells

**Nokuphila Winifred Nompumelelo Simelane, Gauta Gold Matlou and Heidi Abrahamse ***

Laser Research Centre, Faculty of Health Sciences, University of Johannesburg, P.O. Box 17011, Johannesburg 2028, South Africa
* Correspondence: habrahamse@uj.ac.za

## Supporting information

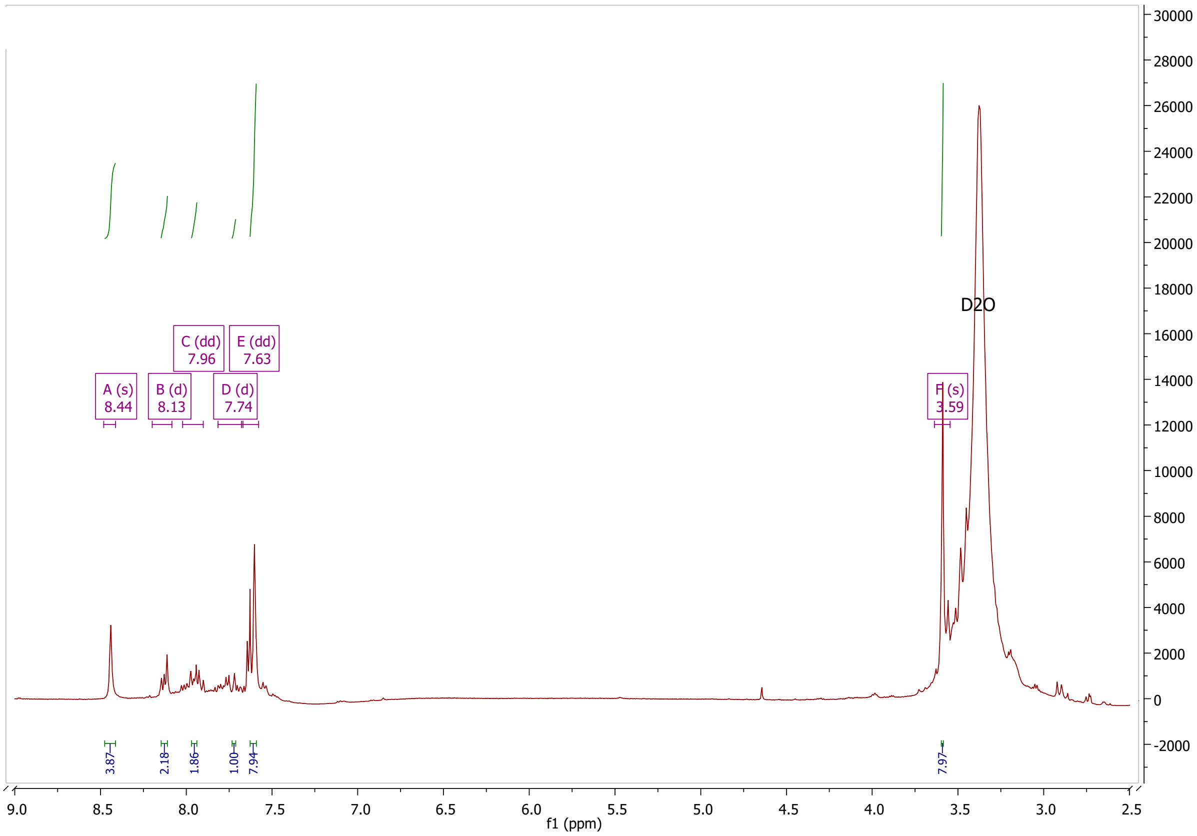


**Figure S1.** 1H NMR spectra of the novel aluminium (III) chloride 2(3), 9(10), 16(17), 23(24)-tetrakis-(sodium 2-mercaptoacetate) phthalocyanines (AlClPcTS41).

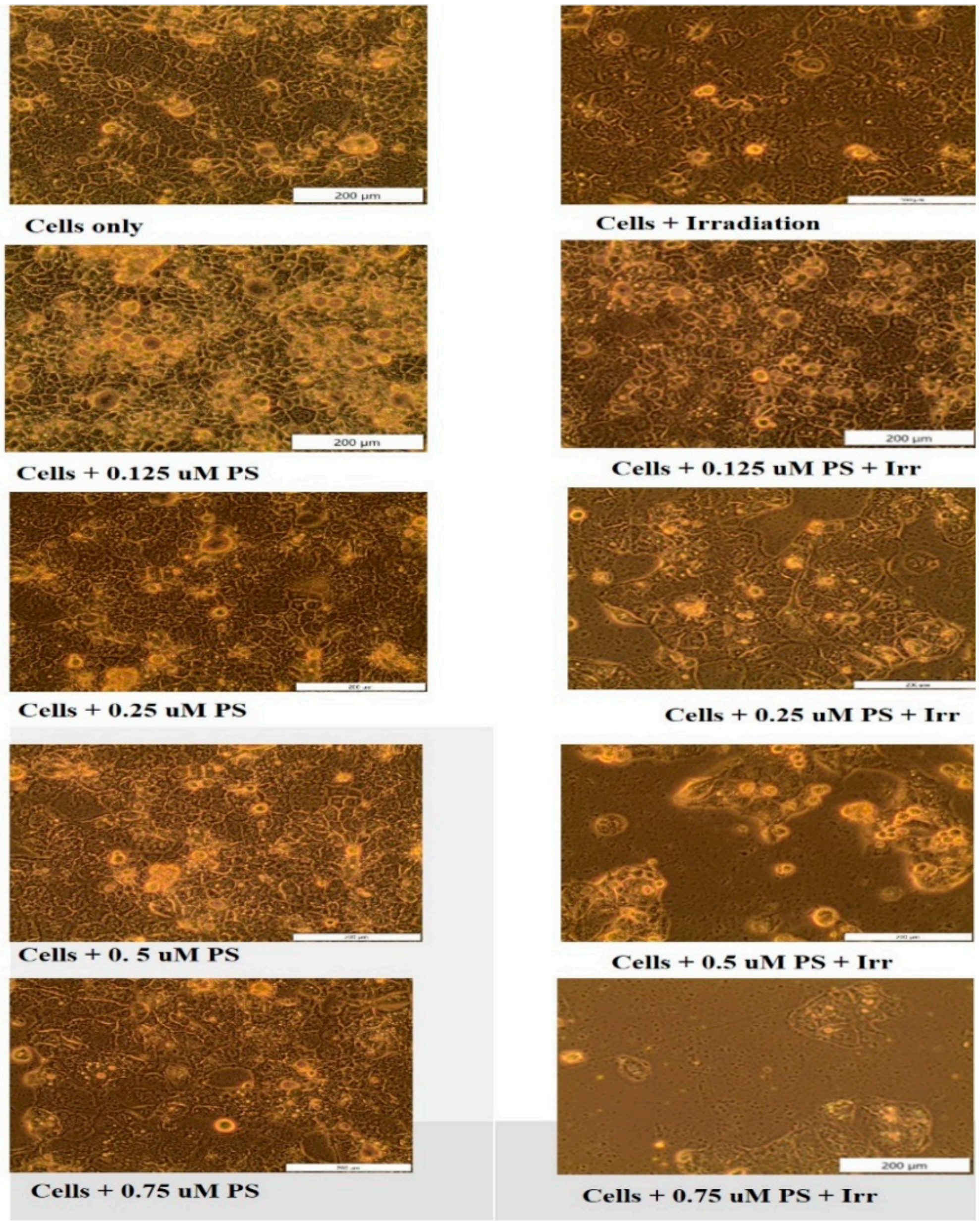


**Figure S2:** Morphology changes observed using an inverted light microscopy, demonstrating (a) control groups (absence of AlClPcTS41 PS or absence of light irradiation) noted unaltered cell structure while (treated group incubated with AICIPcTS41 and light irradiation 636nm and light dose of 10 J cm $^{2}$,distortion in structure was observed, after 24h post irradiation.
